# Avian Influenza Virus Strain Specificity in the Volatile Metabolome

**DOI:** 10.3390/metabo15070468

**Published:** 2025-07-09

**Authors:** Young Eun Lee, Richard A. Bowen, Bruce A. Kimball

**Affiliations:** 1 Monell Chemical Senses Center, Philadelphia, PA 19104, USA; ylee@monell.org; 2 Department of Biomedical Sciences, College of Veterinary Medicine, Colorado State University, Fort Collins, CO 80523, USA; rbowen@rams.colostate.edu

**Keywords:** metabolome, influenza, ducks, diagnostic

## Abstract

Background/Objectives: Outbreaks of highly pathogenic avian influenza virus (AIV) result in significant financial losses and the death or depopulation of millions of domestic birds. Early and rapid detection and surveillance are needed to slow the spread of AIV and prevent its spillover to humans. The volatile metabolome (i.e., the pattern of volatile metabolites emitted by a living subject) represents one such source of health information that can be monitored for disease diagnosis. Indeed, dogs have been successfully trained to recognize patterns of “body odors” associated with many diseases. Because little is known regarding the mechanisms involved in the alteration of the volatile metabolome in response to health perturbation, questions still arise regarding the specificity, or lack thereof, of these alterations. Methods: To address this concern, we experimentally infected twenty mallard ducks with one of two different strains of low-pathogenic AIV (ten ducks per strain) and collected cloacal swabs at various time points before and after infection. Results: Headspace analyses revealed that four volatiles were significantly altered following infection, with distinct profiles associated with each viral strain. The volatiles that differed between strains among post-infection sampling periods included ethylbenzyl ether (*p* = 0.00006), 2-phenoxyethanol (*p* = 0.00017), 2-hydroxybenzaldehyde (*p* = 0.00022), and 6-methyl-5-hepten-2-one (*p* = 0.00034). Conclusions: These findings underscore that AIV-induced changes to the volatile metabolome are strain-specific, emphasizing the need for disease-specific profiling in diagnostic development.

## 1. Introduction

Avian influenza, commonly known as bird flu, is an infectious disease caused by influenza A viruses with strains that range from mild to highly pathogenic [1]. While avian influenza is predominantly found in wild birds, it can also infect domestic poultry, such as chickens, ducks, and turkeys, as well as domestic companion birds like parrots and canaries. In rare cases, certain strains of the virus can be transmitted to humans, raising public health concerns [2]. Avian influenza viruses (AIV) are typically spread through contact with the saliva, nasal secretions, and feces of infected birds. Although most human infections are linked to direct exposure to infected birds or contaminated environments [3], there is concern about the potential for the virus to mutate and become more easily transmissible among humans [4].

Due to its potential to cause both economic losses in the poultry industry and pose serious health risks to humans, AIV infection is closely monitored by public authorities worldwide. Early detection, control measures, and prevention strategies are crucial in managing outbreaks and minimizing the spread of this infectious disease. One of the greatest challenges in mitigating the impact of avian influenza is the virus’s ability to rapidly evolve and spread across vast geographic areas. Given its rapid mutation rates and capacity for RNA segment re-assortment, early detection becomes crucial in controlling the spread of highly pathogenic avian influenza [5]. Without effective early detection, outbreaks can escalate quickly, resulting in both economic losses for the poultry industry and a heightened risk to public health [6].

Using trained animals and chemometric analyses, we previously demonstrated that AIV infection was associated with a marked increase in acetoin (3-hydroxy-2-butanone) in the feces of experimentally infected ducks [7,8]. Acetoin was suggested to result from *Bacillus spp*. acting on elevated levels of pyruvate, which was made available through virus-induced alterations in host cell glycolysis [7]. This key finding was exploited by training ferrets to recognize increased acetoin concentrations (in ratios with 1-octen-3-ol) and then having those trained animals generalize the learned response to feces collected from healthy and infected ducks [8]. The results of that work strongly suggested that acetoin is a repeatable, salient cue for identifying AIV infection in ducks.

The current research emphasized the critical role that the volatile metabolome can play in the early detection of AIV. We highlight the potential of non-invasive monitoring through the identification of volatile biomarkers, which can enable early intervention and help prevent large-scale epidemics. In this context, we explore alterations in the volatile metabolome caused by infection with two strains of AIV, both of which were isolated from wild mallard ducks, and identify areas for concern in the study of the volatile metabolome for infectious disease detection.

## 2. Materials and Methods

### 2.1. Subjects

Twenty farm-raised mallards of mixed gender were used in this study. The birds were group-housed under BSL2+ containment in a room with a children’s swimming pool and ready access to commercial feed. This study was carried out in strict accordance with the recommendations in the Guide for the Care and Use of Laboratory Animals of the National Institutes of Health and the Guide for the Care and Use of Agricultural Animals in Agricultural Research and Teaching of the Federation of Animal Science Societies. Animal procedures were reviewed and approved by the Institutional Animal Care and Use Committee of Colorado State University (#5261).

### 2.2. Experimental Infections

Twenty ducks (ten per virus) were infected a single time with 1 mL of brain–heart infusion broth containing a total 10^6^ pfu/mL of either A/mallard/NY/6750/78 (H2N2) or A/Mallard/MN/346250/00 (H5N2). These two viruses were isolated originally from mallard ducks and therefore represent rational targets for AIV surveillance. The inoculum was distributed ocularly, intranasally, and orally by placing one drop in each eye and dividing the remainder between oral and intranasal introduction. Infection was confirmed by demonstrating viral RNA in cloacal swabs collected 3 days post-inoculation using reverse transcriptase PCR, as described previously [7]. The two groups of ducks were infected with the appropriate strain approximately two months apart. Based on our previous work demonstrating increased fecal acetoin concentration following experimental AIV infection, we calculated that ten subject pairs per strain (pre- and post-infection) would yield power = 0.893 and improved upon the previous design by analyzing samples from multiple time points rather than pooling all pre- and post-infection samples [7].

### 2.3. Sample Collection

For both viral strains, cloacal swabs were taken with wooden-shafted, cotton-tipped applicators on days −2 (two days prior to infection) and 0 (immediately prior to infection), as well as multiple days post-infection (Table 1). The cotton tips were placed in 20 mL glass headspace vials by cutting the wooden shaft with garden shears. A total of 2 mL of 6N guanidine hydrochloride was added to each vial to inactivate the shed virus, and the vials were capped with septa crimp caps. Vials were maintained at −80 °C until they were shipped to the Monell Chemical Senses Center laboratory for headspace analysis.

### 2.4. Headspace Analysis

The collection of headspace volatiles was performed using a TriPlus RSH autosampler (Thermo Scientific, Waltham, MA, USA) for solid-phase microextraction (SPME) with a novel 1.10 mm DVB/C-WR/PDMS Arrow^®^ fiber. A 0.02% aqueous internal standard (IS) solution of acetophenone-*phenyl*-d5 was added to each vial containing a single swab in 2 mL of 6N guanidine hydrochloride (resulting in an approximate IS concentration of 1 ppm). Additional vials, either empty or containing only the internal standard, were periodically analyzed as controls. For analysis, the vial was incubated at 37 °C for 10 min at 500 rpm, followed by a 10 min SPME extraction at 1000 rpm. After extraction, the fiber was inserted into the GC injection port and thermally desorbed in splitless mode at 230 °C for 2 min. The ISQ single-quadrupole gas chromatograph–mass spectrometer (Thermo Scientific, Waltham, MA, USA) was equipped with a 30 m × 0.25 mm ID Stabiliwax^®^ DA-fused silica capillary column (Restek, Bellefonte, PA, USA). The following GC parameters were applied: the oven temperature was initially held at 40 °C for 2 min, and then it was programmed to increase at a rate of 5.0 °C/min to 230 °C, with a 2 min hold at this final temperature. Helium was used as the carrier gas at a constant flow rate of 1.1 mL/min. The mass spectrometer operated with a scan rate of 3.3 Hz, covering a scan range of *m/z* 33–400, with an ion source temperature of 260 °C. A solvent delay time of 5.5 min was applied.

### 2.5. Statistical Analysis

All chromatographic data, representing analyses from both experimental infections, were exported to NetCDF format for baseline correction and peak alignment processing using Metalign software^TM^ [9]. The resulting multivariate data (consisting of all mass spectrometric responses exceeding a defined threshold at each scan event) were then processed using the MSClust tool [10]. MSClust permits the unsupervised determination of chromatographic peaks and yields a single response (corresponding to peak abundance) for each volatile metabolite. Responses from quality control samples were examined to verify that chromatographic processing procedures accounted for retention time shifts between analysis periods and peak alignment. For each sample, peak responses were normalized to the IS peak response recorded for that sample. Multivariate data were first subjected to principal component analysis (PCA), and influence plots were created to visually identify outlier samples exhibiting large residuals or undue leverage using Unscrambler^®^ (version 11.0, CAMO Software; Oslo, Norway). Samples exceeding Hotelling’s T^2^ critical limit were considered outliers and removed from the data set [11].

Because sample collection days (relative to infection date) were not identical between the two virus strains, collection days were grouped into four temporal bins (“Pre”, “Early”, “Mid”, and “Late”), corresponding to known patterns of viral shedding (Table 1). The “Pre” period included all collections made prior to experimental infection. The “Early”, “Mid”, and “Late” periods coincided with the average rise, peak, and decline (respectively) of viral shedding previously observed in experimentally infected ducks [8]. The data were subjected to repeated-measures analysis of variance (ANOVA) to determine if normalized concentrations of any volatile(s) varied according to the viral strain (H2N2, H5N2), period (pre, early, mid, late), or interaction effect between the strain and period using the generalized linear model (PROC GLM) in SAS/STAT version 9.4 [12]. “Volatile” (variable name assigned to the compilation of volatiles in the data set) was the repeated measure. To determine which specific volatiles varied according to strain, period, or interaction effect, univariate analyses were conducted (one for each volatile metabolite). The false discovery rate (FDR) controlling procedure was employed to account for the many univariate ANOVA tests [13]. When warranted, multiple comparisons among strain and period were made using the PDIFF option in SAS. Residuals were plotted to examine their distribution and heteroscedasticity.

## 3. Results

Multiple samples collected from duck #7 (H5N2 infection) were identified as highly influential, based on Hotelling’s T^2^ test, so all sampling periods for this subject were removed from the data. Two additional samples from the H5N2 infection (duck #6, day 2, and duck #8, day −2) were also highly influential and removed as outliers. Repeated measures indicated a significant strain effect (F_1,62_ = 0.4816; *p* < 0.0001), but no other between-subjects effects were significant (Period: F_3,62_ = 0.0169, *p* = 0.4682; Strain*Period: F_3,62_ = 0.04428, *p* = 0.0933). All within-subjects effects were significant: Volatile (F_121,7502_ = 33.73, *p* < 0.0001), Volatile*Strain (F_121,7502_ = 10.70, *p* < 0.0001), Volatile*Period (F_363,7502_ = 0.6973, *p* = 0.0074), and Volatile*Strain*Period (F_363,7502_ = 1.115, *p* < 0.0001), indicating that some volatiles varied among periods, but this effect depended on the virus strain. An examination of residual plots indicated that they were normally distributed, with constant variance among collection periods. Univariate analyses revealed that four volatiles varied between strains and among sample periods (Table 2). No volatiles were only affected by the period main effect, indicating that the two virus strains did not share the same alteration of any single volatile. Importantly, acetoin was not observed in any samples.

**Figure 1:**
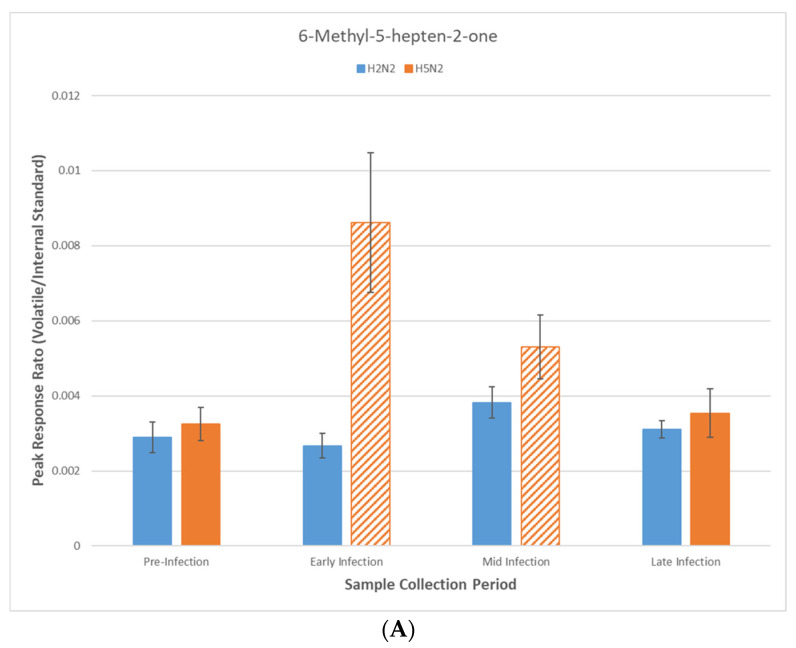

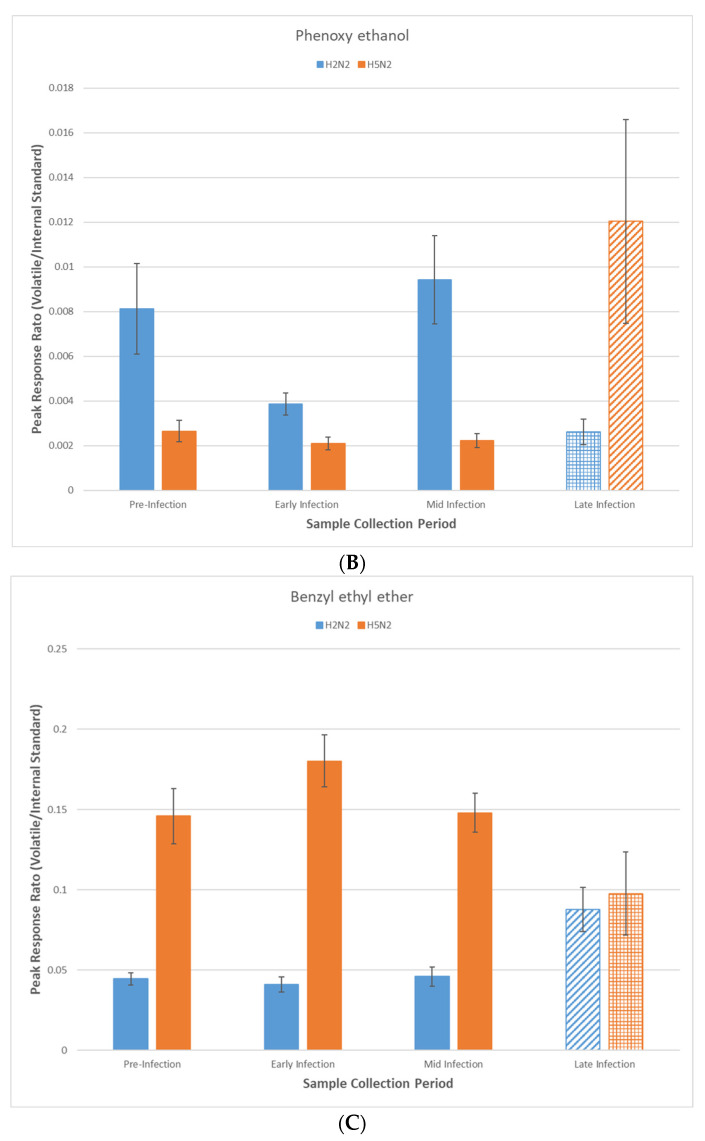

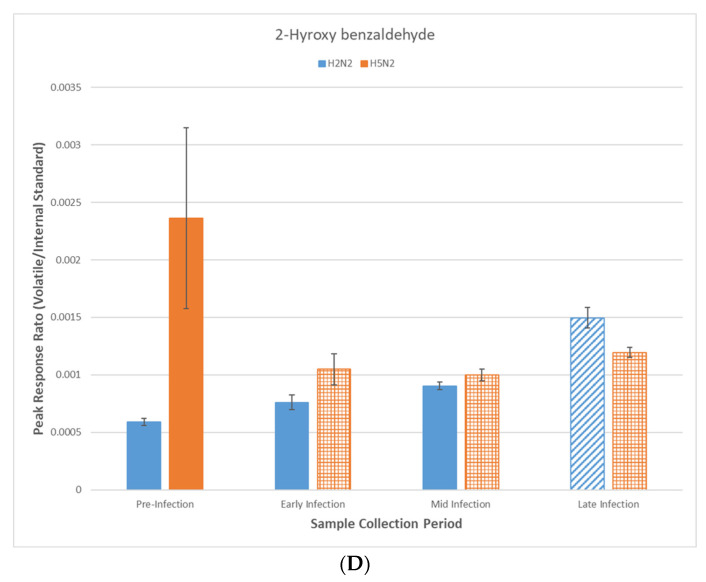
(**A**–**D**) Normalized headspace responses across infection periods for four volatiles with a demonstrated interaction effect between strain and period. Diagonal bars indicate a significant increase (*p* < 0.05) versus the headspace response observed in the pre-infection period for the given virus strain. Crosshatched bars indicate a significant decrease with respect to the pretreatment value.

As compared to the pre-infection period, both increasing and decreasing concentrations of certain volatiles were observed in the headspace of swabs following experimental infection (Figure 1A–D). Among the four volatiles exhibiting a significant interaction effect between strain and period, perhaps only the pattern of 6-methyl-5-hepten-2-one following infection with H5N2 (increase in the early and mid-periods and returning to the pre-infection level during the late period) is suggestive of a potentially useful diagnostic biomarker for H5N2 AIV infection (Figure 1A). Conversely, patterns of increasing or decreasing concentrations of the other three volatiles appear less informative and are often contradictory. For example, the concentrations of 2-phenoxyethanol remain unchanged for both AIV strains from infection through the mid-period. However, in the late period, the concentration of this volatile increased for the H5N2 strain, while it decreased for the H2N2 strain (Figure 1B). Concentrations of ethylbenzyl ether are similarly constant for both AIV strains through the mid-period; however, late-period concentrations inversely change compared to 2-phenoxyethanol (i.e., this volatile increased for H2N2 and decreased for H5N5; Figure 1C). The concentrations of 2-hydroxybenzaldehyde decreased soon after infection with the H5N2 strain and remained lower throughout the length of the experiment. Conversely, 2-hydroxybenzaldehyde followed a pattern similar to that of ethylbenzyl ether for H2N2 infection. Specifically, the concentration remained constant through the mid-period and increased in the late period (Figure 1D).

## 4. Discussion

This study identified four volatiles that varied significantly over time in response to experimental AIV infection in ducks. However, these changes were AIV strain-specific, with no single volatile consistently altered across both viral subtypes (Figure 1A–D). Among these, benzylethyl ether, 2-phenoxyethanol, and 2-hydroxybenzaldehyde are compounds commonly linked to dietary sources or environmental exposure. These volatiles are well-characterized as plant-derived metabolites and are widely used in the flavoring and fragrance industries [14]. Their limited documentation as products of microbial biosynthesis suggests an exogenous origin, likely influenced by host diet or environment.

Conversely, 6-methyl-5-hepten-2-one is a well-established microbial metabolite produced by diverse taxa, including *Staphylococcus*, *Streptomyces*, and *Lentilactobacillus*, as well as various fungal genera, such as *Candida*, *Aspergillus*, and *Penicillium* [15]. The temporal variation observed in this compound, especially during H5N2 infection, may reflect changes in the host-associated microbiota triggered by viral infection. The link between fecal volatiles and the microbiome triggered by vaccination has been demonstrated in skunks [16]. However, a critical caveat is that the H2N2 and H5N2 infection trials were conducted approximately two months apart, under potentially variable dietary, microbial, or environmental conditions. While birds were housed under identical indoor conditions, the potential for seasonal or environmental variation affecting the microbiota or host physiology cannot be ruled out. No environmental data (e.g., temperature or humidity) were recorded during the study, which represents potential limitations in controlling for background variation in volatile emissions. These temporal differences introduce potential seasonal or environmental confounders that could influence volatile profiles independently of infection status. We acknowledge this as a critical limitation, underscoring the challenges of longitudinal surveillance and the need to control for non-viral sources of variability in metabolomics studies.

Cloacal swab analysis, which is primarily used for PCR-based viral detection, may offer additional insights into infection-induced changes in the host’s microbiota. The production of 6-methyl-5-hepten-2-one by multiple microbial taxa suggests that this volatile could serve as an indirect indicator of microbial community restructuring. Microbially derived volatiles have been proposed as non-invasive biomarkers for infection and colonization [17]. However, it is important to note that not all microbial volatiles are pathogenic in origin; even commensal species contribute to the volatile metabolome, particularly under shifting ecological conditions. According to ecological competition theory, infection-induced changes in nutrient availability may favor the proliferation of certain microbial taxa, leading to detectable alterations in both community composition and metabolite production.

Previously, we hypothesized that the microbial production of acetoin—a fermentation product—could serve as a diagnostic marker of AIV infection, mediated through host metabolic alterations. Specifically, AIV was proposed to increase pyruvate availability for acetoin-producing microbes such as *Bacillus* spp. [7]. However, in the present study, acetoin was not detected in any cloacal swab samples, even in samples from ducks that were experimentally infected with the same previously used H5N2 strain. This discrepancy may be attributed to differences in sample type and collection context. While our previous study analyzed intact feces collected from cage floors, the current work relied on cloacal swabs, which do not consistently include visible fecal material. Consequently, the sample matrix available for volatile analysis differed substantially. Cloacal swabs in the current study were treated with 6N guanidine hydrochloride to inactivate the shed virus. Guanidine HCl is a potent chaotrope known to disrupt protein–ligand interactions and may interfere with volatile recovery. Although we did not perform direct controls for its effect in this study, the existing literature suggests that chaotropic agents can significantly alter volatile extraction efficiency [18,19]. During method development, acetoin fortification of swab matrices treated with guanidine HCl demonstrated an unexpectedly high detection threshold (~20 ppm), suggesting that matrix-related suppression or interference may have prevented its detection in this experiment.

Alternatively, the presence of acetoin in the previous study may have been a direct result of gamma irradiation used to inactivate shed virus in fecal samples [20]. Although ionizing radiation is known to generate volatiles, it does not produce acetoin from pyruvate; instead, irradiation typically results in the formation of 2,3-dimethyltartaric acid or acetic acid. The gamma irradiation of lipids has similarly been shown to reduce, rather than increase, ketone levels, including acetoin [21]. More importantly, in a subsequent study with animal biosensors (ferrets) trained to detect AIV infection, the ferrets initially trained to recognize increased concentrations of acetoin in test solutions generalized their response to non-irradiated fecal samples from infected ducks [8]. In other words, irradiation was not necessary to produce the increased acetoin concentrations detected by the ferrets. These findings collectively suggest that gamma irradiation was not responsible for the appearance of acetoin in fecal headspace profiles in the original study.

Finally, it is also possible, while less likely, that microbial acetoin production occurred post-excretion, perhaps facilitated by microbes introduced from the duck’s skin or feathers or the environment. Indeed, several acetoin-producing species, including those commonly found on human skin, are known to persist on dry surfaces and rapidly colonize biological material [22]. While any of these schemes may explain why acetoin was not observed in the headspace of cloacal swabs, the most probable reason was likely a combination of the limited amount of feces collected by swabbing and poor detectability in headspace resulting from the use of guanidine HCl. 

## 5. Conclusions

Taken together, the failure to replicate acetoin detection and the lack of consistent volatile changes across AIV strains emphasize the critical importance of methodological consistency in biomarker discovery studies. Variations in sample type, collection conditions, viral inactivation methods, and even the timing of experiments can profoundly influence the volatile profiles obtained. Future studies should incorporate environmental controls, standardized sample matrices, and parallel testing across viral strains to minimize confounding. Overall, our findings underscore the complexity of the infection-related volatile metabolome and the need for a deeper understanding of host–microbe–metabolite interactions in the context of viral infections.

## Figures and Tables

**Table 1:** Experimental sample collection days relative to the infection event (day 0). Periods coincide with the pattern of viral shedding in ducks [8].

Virus	Pretreatment Period		Early Infection Period		Mid Infection Period		Late Infection Period	
H2N2	−2	0	1	3	5	7	14	21
H5N2	−2	0	2	4	6	8	10	12

**Table 2:** Volatiles subject to experimental effects. Decision criteria (alpha values) were determined according to the method of Benjamini and Hochberg [13].

Volatile Name	Effect Type	*p*-Value	FDR-Adjusted Alpha
Ethylbenzyl ether	Period*Day	*p* < 0.00006	0.00041
2-Phenoxyethanol	Period*Day	*p* = 0.00017	0.00082
2-Hydroxybenzaldehyde	Period*Day	*p* = 0.00022	0.0012
6-Methyl-5-hepten-2-one	Period*Day	*p* = 0.00034	0.0016

## Data Availability

Detailed data summarized in this report will be made available to qualified investigators upon request by contacting the corresponding author.
